# Methanol extract of *Cola nitida* ameliorates inflammation and nociception in experimental animals

**DOI:** 10.1016/j.ynpai.2019.100027

**Published:** 2019-01-21

**Authors:** Lawrence Dayo Adedayo, Alaba Olumide Ojo, Funmileyi Olubanjo Awobajo, Boluwatife Adetoyin Adeboye, James Adedayo Adebisi, Temitope Joshua Bankole, Gideon Opeyemi Ayilara, Olubayode Bamidele, Nimedia Gideon Aitokhuehi, Samuel Adetunji Onasanwo

**Affiliations:** a Neurophysiology Unit, Department of Physiology, Faculty of Basic Medical and Health Sciences, College of Health Sciences, Bowen University, Iwo, Nigeria; b Department of Physiology, Faculty of Basic Medical and Health Sciences, College of Health Sciences, Bowen University, Iwo, Nigeria; c Department of Physiology Faculty of Basic Medical Sciences, College of Health Sciences, University of Ilorin, Nigeria; d Neurosciences and Oral Physiology Unit, Department of Physiology, Faculty of Basic Medical Sciences, College of Medicine, University of Ibadan, Nigeria

**Keywords:** Analgesic, Anti-inflammation, Cola nitida, Carrageenan

## Abstract

Methanol extract of *Cola nitida* (MECN) was evaluated for its anti-inflammatory and analgesic activities using rats and mice. Inflammatory activity of MECN was assessed by carrageenan-induced paw oedema while analgesic activity was evaluated by acetic acid –induced writhing and formalin paw lick test. Histological analyses of the paws were also carried out. There was evaluation of the mechanism(s) of action of MECN using naloxone, a blocker of opioid receptors; atropine, blocker of muscarinic receptors; and propranolol, blocker of beta adrenergic receptors. Findings from the study revealed that MECN has both anti-inflammatory and analgesic properties. These properties were found to be dose dependent with 200 mg/kg of MECN discovered to be the most potent dose. 200 mg/kg was able to cause statistically significant reduction in paw size (p < 0.001) when compared with the carrageenan group. Histological analysis revealed that rats treated with 200 mg/kg of MECN showed no inflammatory cells in the left paw compared to other groups treated with carrageenan. In the formalin test, the number of paw licking was significantly reduced by MECN at 50 mg/kg, 100 mg/kg and 200 mg/kg in both neurogenic and inflammatory pain responses (p < 0.001) even as 200 mg/kg showed the highest percentage inhibition of 98.17% while 100 mg/kg of aspirin showed percentage inhibition of 93.66%. In acetic acid-induced writhing test, 50 mg/kg, 100 mg/kg and 200 mg/kg of MECN produced significant inhibition of writhes when compared with control as highest inhibition is observed in mice that received 200 mg/kg which is similar to aspirin. Administration of propranolol and naloxone was unable to reverse the analgesic function of MECN. However, atropine administration blocked the analgesic function of MECN. This shows that MECN exhibits its analgesic property through cholinergic pathway and not opioid and adrenergic pathways. Phytochemical screening revealed that MECN contains flavonoids, steroids, saponins, tannins, anthraquinines, terpenoids, and alkanoids. These phytochemical contents may thus be responsible for its analgesic and anti-inflammatory properties.

## Highlights

• Methanol extract *Cola nitida* possesses analgesic properties in mice.
• Methanol extract of *Cola nitida* showed anti-inflammatory activity.
• Methanol extract of cola nitida mediates its nociceptive action through cholinergic pathway.
• Opioid and beta adrenergic pathways do not mediate the analgesic potential of *Cola nitida.*

## Graphical abstract

**Figure:**
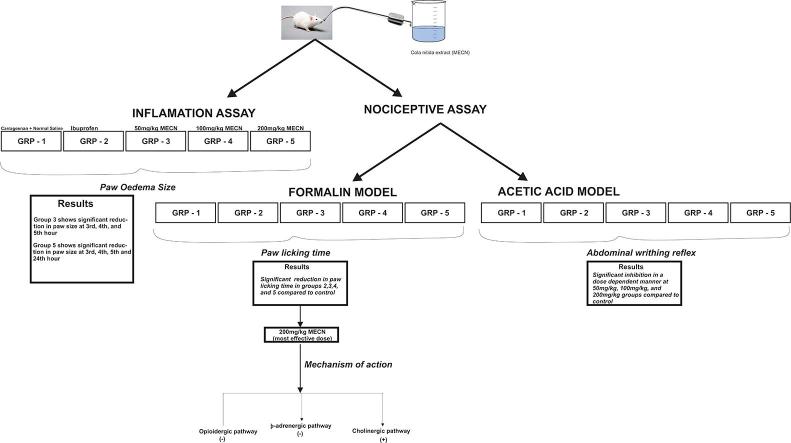

## 1 Introduction

Pain and inflammation is a common reason to seek medical attention due to its high prevalence (Johannes et al., 2010). Acute inflammation in most cases is beneficial to the body as means of protection, however when it becomes chronic, it serves no beneficial purpose as it causes tissue damage and pain. There is still search for potent and effective analgesics despite the progress made in developing pain therapy. Non-steroidal anti-inflammatory drugs (NSAIDs) are drugs usually used for treating pain and inflammation. These drugs exhibit their anti-inflammatory effects through the inhibition of *cyclo*-oxygenase thereby preventing the formation of prostaglandins (Vane and Botting, 1998). Acute and chronic use of most of the pain and inflammation relief medications such as NSAIDs and opioids is associated with many untold side effects and potential interactions with other drugs. Therefore, there is increasing interest in the use of herbal medicine as an alternative or adjuvants for anti-inflammatory and anti-nociceptive agents. NSAIDs are known to cause hepatitis (Dunk et al., 1982), esophageal injury (Wilkins et al., 1984), pulmonary reactions (Aaron and Muttitt, 1982), and musculoskeletal reactions (Ronningen and Langeland, 1979). Furthermore, NSAIDs increase risk of myocardial infarction, (Kearney et al., 2006), erectile dysfunction (Shiri et al., 2006), and adverse effect on the kidney (Schneider et al., 2006).

Prostaglandins synthesized by *cyclo*-oxygenases are the mediators of inflammatory and pain. Their synthesis is induced by inflammatory stimuli such as cytokines and growth factors (Ballou et al., 2000). These chemicals induce pain through sensitization of nociceptors (England et al., 1996). Therefore, blocking of prostaglandin synthesis has been the method for alleviating pain.

*Cola nitida* is a seed consumed mostly by elderly people. The seed had traditional, social, and medicinal importance in different cultures of the world (Dewole et al., 2013). *Cola nitida* is known as kola nut in English language, “obi” in Yoruba language. In Yoruba culture, it is used as gesture of friendship and is vital in traditional ceremonies as symbol of peace and wishes for good will. It is the fruit of evergreen kola tree that is native to Africa, in its tropical rainforests (Adisa et al., 2010). *Cola nitida* is consumed by many to withstand fatigue and keep them awake. This physiological effect has been attributed to the caffeine content of kola nut (Chukwu et al., 2006).

The use of natural products for medicinal purposes in primary health care is recognized by the World Health Organisation (WHO, 2005). Also, there are many drugs such as quinine, reserpine, morphine, strychnine, and so on that were isolated from plants (Farnsworth and Bingel, 1997).

There have been studies on *Cola nitida*. Dewole et al (2013) did the proximate and phytochemical analysis of *Cola nitida*. *Cola nitida* was found to have alkaloid, phenol, tannin, flavonoid, and saponin. The effect of *Cola nitida* on reproductive hormones was studied by Adisa et al. (2010). Also, Adesanwo et al. (2017) reported the antimicrobial and antioxidative properties of *Cola nitida*. There is however paucity of studies on anti-inflammatory, nociception and mechanism(s) of *Cola nitida* in rodents.

Therefore, the aim of this study is to investigate the anti-inflammatory and anti-nociceptive activities of methanol extract of *Cola nitida.*

## 2 Materials and method

### 2.1 Chemicals and drugs

All chemicals/reagents were purchased from Sigma Chemical Co. (Germany), unless otherwise stated. Glacial acetic acid, formaldehyde, carrageenan, were purchased from S.D. Fine Chemical Pvt. Ltd. (Mumbai, India). The extract was dissolved in tween 20. All other chemical were of analytical graded. Aspirin, ibuprofen, naloxone hydrochloride, atropine sulphate, and propranolol were purchased from Alpha Pharmacy, Lagos, Nigeria.

### 2.2 Animals

Female Wistar rats weighing 100–120 g and aged 8–10 weeks and Albino mice (15–23 g) aged 10–12 weeks were acclimatized for two weeks before the initiation of the experiment, and maintained under standard nutritional and environmental condition (12-hour light/day cycle). They were purchased from Animal House, Bowen University, Iwo. They had free access to standard pellet diet and water *ad libitum*. The animals were kept in separate cages according to their groups. Animals were deprived of food for 16hrs before experimentation in analgesic acid model to prevent interaction of food with the visceral writhing test. All procedures involving the use of animals in this study complied with the guiding principles for research involving animals as recommended by the declaration of Helsinki and the Guiding principles in the care and use of animals.

### 2.3 Plant materials

Fresh seeds of *Cola nitida* were collected in Odori market, Iwo. The identification and authentication of the seeds was carried out at Department of Botany, Obafemi Awolowo University, Ile-Ife, Nigeria on the 16th March 2016 with voucher number: FHI.17512.

### 2.4 Preparation of extracts

The seeds were air dried at room temperature and ground to fine powder. It was then extracted, using gradient extraction method. 3.56 kg of grinded *Cola nitida* powde*r* was soaked in several beakers, in 8.365 L of methanol for 72 h at room temperature after which it was filtered using 150 mm Whatmann filter paper then the residue was squeezed to yield more filtrate. It was then evaporated to dryness by a rotary evaporator at 40 °C. Percentage yield of *Cola nitida* was 3.79%. The yield weighed 240 g.

### 2.5 Evaluation of anti-inflammatory activity in rats

Before the experiment began, the paw sizes of the left hind paw of the rats were determined using the automatic vernier caliper. The rats were then divided into five groups containing six animals each. Normal Saline 10 ml/kg, ibuprofen 10 mg/kg (Reddy et al., 2012), or *Cola nitida* (doses of 50, 100 and 200 mg/kg b.w) was administered orally one hour before 0.1 ml of 1% carrageenan was delivered into the sub-plantar surface of the left hind paw of the rats in the five groups. The extract and ibuprofen were administered using Tween 20 and normal saline as vehicle. The paw sizes were measured at 0, 1, 2, 3, 4, 5, 6 h, and also 24 h after injection of carrageenan. After the 24 h of measuring the paw size for each group, the rats were then euthanized and the paw tissues were harvested for histological evaluation

### 2.6 Evaluation of anti-nociceptive activity in mice

#### 2.6.1 Formalin-induced nociception model

The procedure described by Hunskaar and Hole (1987) was followed. There were five groups containing 6 mice each. Group 1 received 10 ml/kg normal saline orally while group 2, 3, 4 received 50,100 and 200 mg/kg of *Cola nitida* extract respectively through oral administration. Group 5 received 100 mg/kg of aspirin orally (Adedayo et al, 2017). One hour later, 20 μl of 1% formalin was injected subcutaneously into the dorsal surface of the left hind paw of the mice in all the groups using a micro syringe. The mice were then placed in a transparent 30 × 30 × 30 cm to allow unobstructed view of the mice. The response was bi-phasic. There was an initial, acute nociceptive response which peaked at 5 min after formalin injection (0–5) minutes indicated the (early phase) of the paw licking nociceptive response while the (late phase) of the response followed between 20 and 30 min after formalin injection. The early and late phases represented the neurogenic and inflammatory pain responses respectively (Hunskaar and Hole, 1987). In each phase, percentage inhibition of the mean paw licking time of the treated groups as compared with the control group is indicative of analgesia.

$$\frac{\%\;\text{inhibition}=\text{mean}\;\text{response}\left(\text{control}\right)-\text{mean}\;\text{response}\;\left(\text{treated}\right)}{\text{Mean}\;\text{response}\left(\text{control}\right)}\times 100$$

#### 2.6.2 Acetic acid –induced writhing model in mice

Siegmund et al. (1957) method was used to assess anti-nociceptive activity in the mice. The experimental mice were divided into five groups of five mice per group. Control group received normal saline. Group 2, 3, 4 received 50, 100, 200 mg/kg of *C.nitida*. Group five received 100 mg/kg of aspirin (Adedayo et al., 2017). One hour after treatment, painful writhing was induced in the mice in all experimental groups by injecting 0.2 ml of 3% acetic acid solution intraperitoneally (abdominal region) and then placed in an observation chamber. The number of writhes was observed between 5 and 15 min. The data were collected and computed according to the following formula:

$$\frac{\%\;\text{inhibition}=\text{mean}\;\text{writhes}\left(\text{control}\right)-\text{mean}\;\text{writhes}\left(\text{treated}\right)}{\text{Mean}\;\text{writhes}\left(\text{control}\right)}\times 100$$

### 2.7 Pain mechanism

To understand the mechanism of action of *Cola nitida*, the mice were grouped into three different models containing five mice per group in each model, with each model containing five groups. Mice in group 5 of each model were pre-treated with three different antagonist drugs (Naloxone, Atropine, and Propranolol). 2 mg/kg doses were used for these antagonist drugs as obtained from similar studies (Luciano et al., 2012, Afify et al., 2017) Fifteen minutes later, the mice in each group were treated with distilled water, *Cola nitida*, or aspirin. One hour after, pain was induced in all the groups using the formalin. Pain responses were measured using the procedure described above.

The grouping was done as follows:

For Naloxone;

• Group I was administered with Distilled Water (10 ml/kg)
• Group II was administered with *Cola nitida* (200 mg/kg)
• Group III was administered with Distilled Water and *Cola nitida* (200 mg/kg)
• Group IV was administered with Aspirin (100 mg/kg)
• Group V was administered with Naloxone (2 mg/kg) and *Cola nitida* (200 mg/kg)

For Atropine;

• Group I was administered with Distilled Water (10 ml/kg)
• Group II was administered with *Cola nitida* (200 mg/kg)
• Group III was administered with Distilled Water and *Cola nitida* (200 mg/kg)
• Group IV was administered with Aspirin (100 mg/kg)
• Group V was administered with Atropine (2 mg/kg) and of *Cola nitida* (200 mg/kg)

For Propranolol;

• Group I was administered with Distilled Water (10 ml/kg)
• Group II was administered with *Cola nitida* (200 mg/kg)
• Group III was administered with Distilled Water and *Cola nitida* (200 mg/kg)
• Group IV was administered with Aspirin (100 mg/kg)
• Group V was administered with Propranolol (3 mg/kg) and *Cola nitida* (200 mg/kg)

### 2.8 Preliminary phytochemical screening

The methanol extract of *Cola nitida* was subjected to preliminary screening for various active phytochemical constituents such as alkaloids, cardenoldes, anthraquinone, saponins, flavonoids and tannins. The phytochemical screening can be carried out using Drangenduff’s reagent, Meyer’s reagent or Wanger’s reagent for alkaloids; keller-killiani reagent or kedde reagent for cardenoldes; chloroform/ammonia test for free anthraquinone; frothing test for saponins; ferric chloride test for tannins and DPPH for flavonoids (Trease and Evans, 1999).

### 2.9 Histological analysis

Paw tissues were harvested and fixed in 10% formaldehyde solution, dehydrated in graded alcohol and later embedded in paraffin wax. The tissues were then cut into sections by using a microtome and were subsequently stained with hematoxylin-eosin (H&E). The slides were examined under a light microscope with the photomicrographs taken.

### 2.10 Statistical analysis

Data were expressed as mean ± standard error of the mean. The data obtained were analysed using one-way ANOVA by Graphpad Prism 7.0. *Post-hoc* testing was performed for inter-group comparison using the Bonferroni test. Results were considered to be significant at p < 0.05.

## 3 Result

### 3.1 Phytochemical result of MECN

Table 1 shows the phytochemical analysis the methanol extract of Cola nitida. MECN contains flavonoids, steroids, saponins, tannins, anthraquinines, terpenoids, and alkanoids. MECN does not contain cardiac glycoside.

**Table 1:** Phytochemical screening result of the methanol extract of cola nitida.

BIOACTIVE COMPUNDS	TEST	OBSERVATION	INFERENCE
ALKALOIDS	Mayer’s reagent test	Colour change	Alkaloids present (+)
ANTHRAQUINONES	Chloroform/Ammonia test	Colour change	Anthraquinones present (+)
CARDIAC GLYCOSIDES	Kedde’s test	No colour change	Cardiac glycosides absent (−)
FLAVONOIDS	Lead acetate test	Colour change	Flavonoids present (++)
SAPONINS	Froth test	Persistent frothing	Saponins present (++)
STEROIDS	Liebermann Burchard’s test	Colour change	Steroids present(+)
TANNINS	Ferric chloride test	Colour change	Tannins present (++)
TERPENOIDS	Chloroform/Sulfuric acid test	Colour change	Terpenoids present (+)

### 3.2 Effect of MECN on carrageenan-induced paw oedema

Table 2 shows the effect of MECN on the paw sizes of the animals. In the group administered carrageenan with normal saline, the paw size increased progressively and significantly from 2.94 ± 0.15 mm before the induction of carrageenan to 5.29 ± 0.26 mm. At the 5th hour, the paw sizes in carrageenan group begin to reduce. This shows the potency of the carrageenan. The paw sizes of the group treated with 50 mg/kg of MECN began to increase after administration of carrageenan but by the second hour, the paw sizes begin to show statistically significant reduction (P < 0.05) when compared with the carrageenan group. The group treated with 100 mg/kg of MECN showed decrease in the paw size but not statistically significant (P < 0.05) when compared with the carrageenan group. 200 mg/kg MECN group showed statistically significant reduction (P < 0.01) in the paw size beginning from the 4th hour. In the group treated with ibuprofen, the paw sizes begin to reduce at the 1st hour. At the fourth however, 50 mg/kg and 200 mg/kg showed better inhibition (P < 0.01) compared to ibuprofen group.

**Table 2:** Effect of MECN on Carrageenan-induced paw oedema.

	Paw Size (mm)				
Reaction Time	Carrageenan Group + normal saline 10 ml/kg	Ibuprofen 10 mg/kg Reference Group 10 mg/kg	50 mg/kg of *Cola nitida*	100 mg/kg of *Cola nitida*	200 mg/kg of *Cola nitida*
0 h	5.29 ± 0.26	4.74 ± 0.21	5.36 ± 0.23	5.53 ± 0.31	4.93 ± 0.25
1 h	5.71 ± 0.39	4.08 ± 0.25^c^	5.07 ± 0.24	5.06 ± 0.23	4.82 ± 0.08
2 h	5.16 ± 0.31	4.00 ± 0.15^b^	4.44 ± 0.06	4.91 ± 0.19	4.43 ± 0.17
3 h	5.14 ± 0.36	4.05 ± 0.14^b^	4.19 ± 0.07^a^	4.70 ± 0.23	4.17 ± 0.18 ^a^
4 h	5.38 ± 0.29	4.47 ± 0.18^b^	4.00 ± 0.12^b^	4.60 ± 0.24	4.21 ± 0.14^b^
5 h	4.87 ± 0.21	3.94 ± 0.10^b^	3.93 ± 0.08^b^	4.40 ± 0.27	4.04 ± 0.14 ^a^
6 h	4.35 ± 0.15	3.69 ± 0.11 ^a^	3.89 ± 0.07	4.37 ± 0.25	3.83 ± 0.12
24 h	3.95 ± 0.09	3.55 ± 0.16	3.73 ± 0.13	3.66 ± 0.10	3.16 ± 0.11^b^

Values are expressed as Mean ± S.E.M. Significant difference exist at ^a^p < 0.05, ^b^p < 0.01, ^c^p < 0.001 compared with the carrageenan group. Carrageenan group received 10 ml of normal saline and 0.1 ml of 1% carrageenan (n = 6). 50 mg/kg MECN group received 50 mg/kg of MECN and 0.1 ml of 1% carrageenan (n = 6). 100 mg/kg MECN group received 100 mg/kg of MECN and 0.1 ml of 1% carrageenan (n = 6). 200 mg/kg MECN group received 200 mg/kg of MECN and 0.1 ml of 1% carrageenan (n = 6). Ibuprofen group received 10 mg/kg of ibuprofen and 0.1 ml of 1% carrageenan (n = 6).

**Fig. 1:**
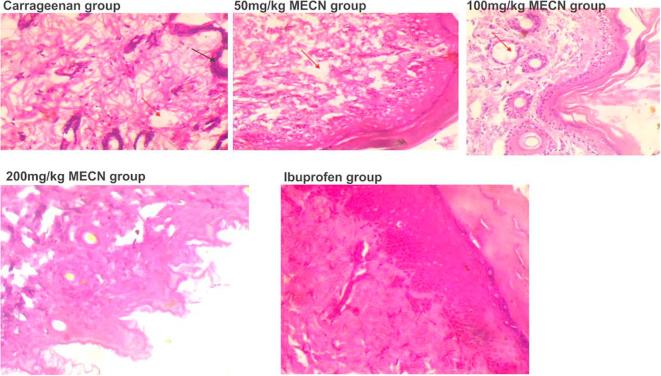
Carrageenan group received 10 ml of normal saline and 0.1 ml of 1% carrageenan (n = 6). 50 mg/kg MECN group received 50 mg/kg of MECN and 0.1 ml of 1% carrageenan (n = 6). 100 mg/kg MECN group received 100 mg/kg of MECN and 0.1 ml of 1% carrageenan (n = 6). 200 mg/kg MECN group received 200 mg/kg of MECN and 0.1 ml of 1% carrageenan (n = 6). Ibuprofen group received 10 mg/kg of ibuprofen and 0.1 ml of 1% carrageenan (n = 6). Carrageenan group showed presence of inflammatory edema (red arrows) and necrotic cells (black cells) (H & E, 400×). 50 mg/kg MECN group shows paw tissue with moderate amounts of mononuclear inflammatory cells enmeshed between the dense connective tissue of the dermis with mild inflammatory edema (red arrow) (H & E, 400×). 100 mg/kg MECN group have paw tissue with moderate amounts of polymorphonuclear inflammatory cells just below the dermis are perifollicular regions (H & E, 400×). 200 mg/kg MECN group have dermis consisting of closely-packed dense connective tissue with the absence of inflammatory cells (H&E, 400×). The ibuprofen group have paw tissue closely-packed dense fibrous connective tissue with no inflammatory cells (H & E, 400×).(For interpretation of the references to colour in this figure legend, the reader is referred to the web version of this article.)

Fig. 1 shows the Haematoxylin and Eosin stained paw tissue in each group.

### 3.3 Effect of MECN on formalin-induced nociception

**Fig. 2:**
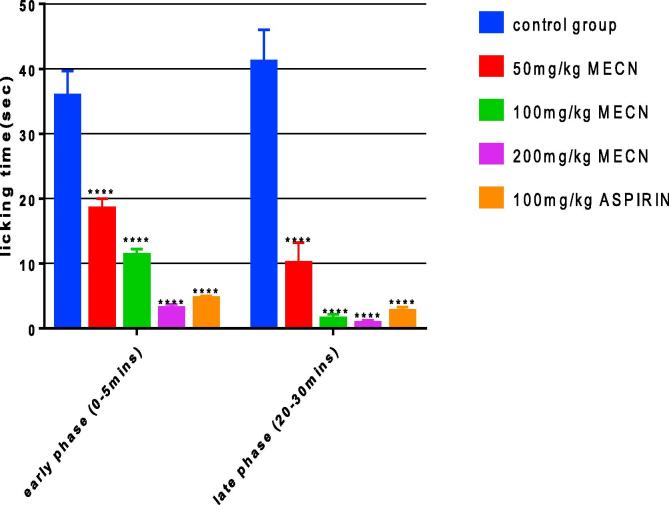
Effect of doses of MECN on formalin-induced paw licking time in mice. Values were expressed as mean ± SEM, N = 6 in each group. ^****^p < 0.001, compared to control group. Control group received 10 ml of normal saline and 20 μl of 1% formalin (n = 6). 50 mg/kg MECN group received 50 mg/kg of MECN and 20 μl of 1% formalin (n = 6). 100 mg/kg MECN group received 100 mg/kg of MECN and 20 μl of 1% formalin (n = 6). 200 mg/kg MECN group received 200 mg/kg of MECN and 20 μl of 1% formalin (n = 6). Aspirin group received 100 mg/kg aspirin and 20 μl of 1% formalin (n = 6).

Fig. 2 showed the analgesic effect of graded doses (50,100,200 mg/kg) of MECN and 100 mg/kg of aspirin on time of paw licking in formalin induced paw licking in mice. There was significant reduction (P < 0.001) of paw licking time in both early (0–5 mins) and late phase (20–30 mins) in groups treated with doses of MECN compared with the control group that received only normal saline and formalin. The control group spent more time licking their paws than the treated groups in both phases. In the MECN and aspirin groups, the licking time was longer in the early phase which represents neurogenic pain than in the late phase which represent inflammatory pain. In the late phase, 200 mg/kg showed the highest percentage inhibition of 98.17% which is statistically higher (p < 0.001) than the inhibition produced by 100 mg/kg of aspirin (93.66%).

### 3.4 Effect of MECN on acetic acid –induced writhing model in mice

Table 3 shows the effect of MECN on Acetic acid-induced writhing model in mice. Number of writhes displayed by group treated with 200 mg/kg of MECN (0 ± 0) was significantly lower (p < 0.0001) compared with the control group treated with acetic acid only. Percentage inhibition increases in a dose dependent manner with values of 71.7%, 84.39%, and 100% for 50,100 and 200 mg/kg group of MECN respectively. The 200 mg/kg group of MECN produced significant inhibition (P < 0.0001) which is similar to the group that were treated with aspirin.

**Table 3:** Effect of MECN on Acetic acid –induced writhing model in mice.

Groups	Mean number of writhing	% Inhibition
Control group	41 ± 3.96	
50 mg/kg MECN group	11.6 ± 0.75^d^	71.7%
100 mg/kg MECN group	6.4 ± 1.86^d^	84.39%
200 mg/kg MECN group	0 ± 0^d^	100%
Reference group (Aspirin)	0 ± 0^d^	100%

Values are expressed as mean ± SEM, N = 6 in each group, ^d^p < 0.0001 vs control. Control group received 10 ml of normal saline and 20 μl of 1% formalin (n = 6). 50 mg/kg MECN group received 50 mg/kg of MECN and 0.2 ml of 3% acetic acid solution. 100 mg/kg MECN group received 100 mg/kg of MECN and 0.2 ml of 3% acetic acid solution. 200 mg/kg MECN group received 200 mg/kg of MECN and 0.2 ml of 3% acetic acid solution. Aspirin group received 100 mg/kg aspirin and 0.2 ml of 3% acetic acid solution.

### 3.5 Effect of naloxone on anti-nociceptive action of *Cola nitida* extract

Table 4 shows the effect of naloxone on anti-nociceptive action of *Cola nitida* extract. There was significant reduction (P < 0.0001) in paw licking time in both early (0–5 mins) and late phase (20–30 mins) in groups 2, 3, and 4 treated with 200 mg/kg compared with the control group that received acetic acid only. Group 4 pretreated with naloxone also showed statistically significant reduction (P < 0.0001) in paw licking time in both early and late phase when compared with the control group treated with acetic acid only. This outcome shows that the mechanism of anti-nociceptive action of MECN is not through the opioid receptors.

**Table 4:** Effect of Naloxone on anti-nociceptive action of *Cola nitida* methanol extract.

GROUP	EARLY PHASE (0–5 MINUTES)	LATE PHASE (20–30 MINUTES)
GROUP I – CONTROL (Distilled Water 10 ml/kg + formalin)	28.8 ± 0.374	25 ± 0.316
GROUP II (*Cola nitida* 200 mg/kg + formalin)	3.6 ± 1.327^d^	1 ± 0.548^d^
GROUP III (Distilled Water 10 ml/kg + *Cola nitida* 200 mg/kg + formalin)	3 ± 0.548^d^	1 ± 0.316^d^
GROUP IV (Naloxone 2 mg/kg + *Cola nitida* 200 mg/kg + formalin)	2.6 ± 0.748^d^	0.8 ± 0.374^d^
GROUP V (Aspirin 200 mg/kg + formalin)	3 ± 0.316^d^	1.4 ± 0.245^d^

Values were expressed in seconds as: Mean ± SEM, n = 6 per group. ^d^p < 0.0001 vs control.

Group 1 received 10 ml/kg of distilled water and 20 μl of 1% formalin (n = 6). Group 2 received 200 mg/kg of MECN and 20 μl of 1% formalin (n = 6). Group 3 received 10 ml/kg distilled water, 200 mg/kg of MECN and 20 μl of 1% formalin (n = 6). Group 4 received 2 mg/kg of Naloxone, 200 mg/kg of MECN and 20 μl of 1% formalin (n = 6). Aspirin group received 200 mg/kg aspirin and 20 μl of 1% formalin (n = 6).

### 3.6 Effect of atropine on anti-nociceptive action of *Cola nitida* methanol extract

**Fig. 3:**
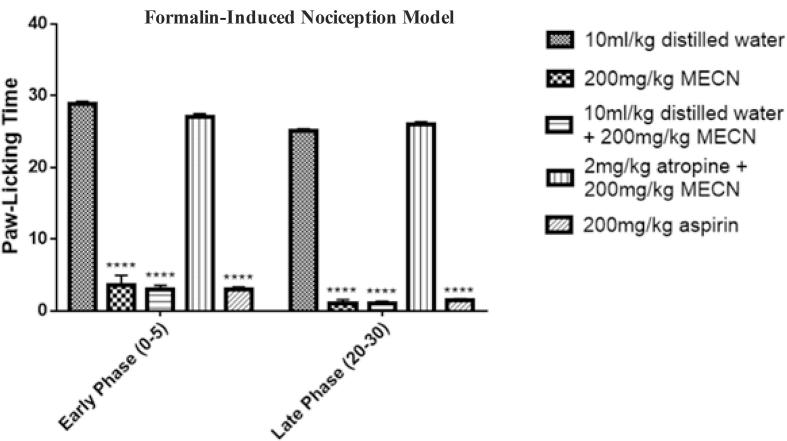
Effect of Atropine on anti-nociceptive action of *Cola nitida* methanol extract. Values are expressed as mean ± SEM, n = 6 in each group. ^****^P < 0.001, compared with the control. Group 1 received 10 ml/kg of distilled water and 20 μl of 1% formalin (n = 6). Group 2 received 200 mg/kg of MECN and 20 μl of 1% formalin (n = 6). Group 3 received 10 ml/kg distilled water, 200 mg/kg of MECN and 20 μl of 1% formalin (n = 6). Group 4 received 2 mg/kg of atropine, 200 mg/kg of MECN and 20 μl of 1% formalin (n = 6). Aspirin group received 200 mg/kg aspirin and 20 μl of 1% formalin (n = 6).

Fig. 3 shows the effect of atropine on the anti-nociceptive action of *Cola nitida* extract. There was significant reduction (p < 0.0001) in paw licking time in both early (0–5 mins) and late phase (20–30 mins) in the all the groups treated with 200 mg/kg MECN compared with the control group that received only distilled water. Group 5 pretreated with atropine however showed no statistically significant reduction (P < 0.05) in paw licking time in both early and late phase when compared with the control group. This result suggested that cholinergic pathway plays a significant role in mediating the anti-nociceptive action of *Cola nitida*.

### 3.7 Effect of Propranolol on anti-nociceptive action of *Cola nitida* methanol extract

When compared with the control group that received formalin and distilled water, all the treated groups showed statistically significant reduction (P < 0.001) in paw licking time in both early and late phase. Group 4 pretreated with propranolol also showed statistically significant reduction (P < 0.05) in paw licking time in both early and late phase when compared with the control group. The result showed that *Cola nitida* does not exert its anti-nociceptive action through the beta-adrenergic pathway (Table 5).

**Table 5:** Effect of Propranolol on anti-nociceptive action of *Cola nitida.*

GROUP	EARLY PHASE (0–5 MINUTES)	LATE PHASE (20–30 MINUTES)
GROUP I – CONTROL (formalin + Distilled Water 10 ml/kg)	28.8 ± 0.374	25 ± 0.316
GROUP II (formalin + *Cola nitida* 200 mg/kg)	3.6 ± 1.327^d^	1 ± 0.548^d^
GROUP III (formalin + Distilled Water 10 ml/kg & *Cola nitida* 200 mg/kg)	3 ± 0.548^d^	1 ± 0.316^d^
GROUP IV (formalin + Propranolol 2 mg/kg & *Cola nitida* 200 mg/kg)	2.8 ± 0.663^d^	0.8 ± 0.374^d^
GROUP V (formalin + Aspirin 200 mg/kg)	3 ± 0.316^d^	1.4 ± 0.245^d^

Values were expressed in seconds as: Mean ± SEM, n = 6 per group. dp < 0.0001 vs control. Group 1 received 10 ml/kg of distilled water and 20 μl of 1% formalin (n = 6). Group 2 received 200 mg/kg of MECN and 20 μl of 1% formalin (n = 6). Group 3 received 10 ml/kg distilled water, 200 mg/kg of MECN and 20 μl of 1% formalin (n = 6). Group 4 received 2 mg/kg of propranolol, 200 mg/kg of MECN and 20 μl of 1% formalin (n = 6). Aspirin group received 200 mg/kg aspirin and 20 μl of 1% formalin (n = 6).

## 4 Discussion

The aim of the study is to investigate the anti-nociceptive and anti-inflammatory properties of *Cola nitida*. The findings from the study revealed that methanol extract of *Cola nitida* possesses anti-inflammatory and anti-nociceptive properties. These properties were found to be dose dependent with 200 mg/kg of MECN discovered to be the most potent dose. Also, the phytochemical screening revealed that MECN contains flavonoids, steroids, saponins, tannins, anthraquinines, terpenoids, and alkanoids. This is similar to the findings of Dewole et al. (2013). These active agents may be responsible for the analgesic and anti-inflammatory properties of MECN.

Carrageenan induced inflammation has been used for inflammatory studies to test the potency of anti-inflammatory agents (Badole et al., 2012). Although only female animals were used in this experiments, there are however the possibilities of the sex imparting on the pain responses recorded and the result may change if male animals were used (Casimir, 2011). Other authors have reported that responses to inflammatory pain may seems to be affected by sex (Casimir et al., 2010)

Carrageenan injection produces paw oedema in a biphasic fashion. Oedema in the first phase is mediated by histamine, serotonin and bradykinin while the second phase (3–5 h) is mediated by prostaglandins (Marrassini et al., 2010). 50 mg/kg, 100 mg/kg and 200 mg/kg doses of MECN inhibited edema from the first hour till the last hour of the experiment. 50 mg/kg showed statistically significant reduction in paw sizes at the 3rd, 4th and 5th hour when compared with carrageenan group. 100 mg/kg of MECN showed no statistical significant reduction when compared with carrageenan group. 200 mg/kg showed significant reduction (p < 0.05) in paw size at the 3rd, 4th, 5th, and 24th hour when compared with the carrageenan group. The effect of MECN can be attributed to the inhibition of release of prostaglandins. The histological analysis of the left hind paw of animals further proved the anti-inflammatory activity of MECN. Rats treated with 200 mg/kg of MECN showed no inflammatory cells in the left paw while those treated with 50 mg/kg showed presence of scanty amounts of mononuclear inflammatory cells. The presence of mononuclear phagocytes is to reduce inflammation through phagocytosis thereby promoting tissue repair (Wynn et al., 2013) The carrageenan group however had presence of mononuclear cells enmeshed in the dense connective tissue of the dermis. 100 mg/kg group had moderate amounts of polymorphonuclear inflammatory cells in the perifollicular regions and thus the paw size decrease was not statistically significant compared to the carrageenan group. The ibuprofen group had absence of inflammatory cells and thus showed significant reduction in paw size compared to the carrageenan group.

Formalin test has been used as a model for localized inflammatory pain and tonic pain (Coderre et al., 1990, Hong and Abbott, 1994). There are two phases in the formalin test. The early phase (0–5 mins) represents neuropathic pain caused by the activation of C-fibre due to the peripheral stimulus by formalin. The late phase (15–30 mins) represents inflammatory pain caused by the release of serotonin, histamine, bradykinin and prostaglandins (Milano et al., 2008). The results showed that the number of paw licking was significantly reduced (p < 0.0001) by MECN at 50 mg/kg, 100 mg/kg and 200 mg/kg in both neurogenic and inflammatory pain responses in a dose dependent manner. The effect of MECN is more pronounced in the late phase. The result suggested that MECN may be exhibiting its anti-inflammatory effect by preventing the peripheral release of inflammatory mediators such as serotonin, prostaglandins, bradykinin. 200 mg/kg showed the highest percentage inhibition of 98.17% which is even better than inhibition produced by 100 mg/kg of aspirin (93.66%). Thus, MECN’s analgesic activity probably resulted from peripheral action.

Acetic acid-induced writhing test is also a model that had been used to test the potency of analgesic agents (Vyklicky, 1979). The test showed that oral administration of 50 mg/kg, 100 mg/kg and 200 mg/kg of MECN produced significant inhibition of writhes. The highest inhibition (100%) is observed in group that received 200 mg/kg which is similar to aspirin. Lowering the number of writhes is caused by the inhibition of prostaglandin synthesis (Loganayaki et al., 2012).

We proceeded further to investigate the mechanism of action of the analgesic property of MECN. Naloxone and propranolol are non-selective opioid receptors blocker and selective beta adrenergic blocker respectively that have been used in studies that evaluate mechanisms of action of analgesic compounds (Berrocoso et al., 2004, Marchand et al., 2003). Propranolol failed to reverse analgesic effect of MECN in both early and late phase of formalin-model group when compared with the group that received distilled water and 200 mg/kg MECN. Analgesic effect of MECN was also not reversed by naloxone in both early phase and late phase. The formalin-model group treated with atropine and 200 mg/kg MECN showed no reduction in paw licking time in both early and late phase as found in the group treated with 200 mg/kg MECN and distilled water but no atropine. This shows that MECN exhibited the analgesic effect by passing through the cholinergic pathway but not beta-adrenergic and opioid pathways. Stimulation of neuronal nicotinic receptors has been known to produce analgesic effects both in human and experimental animals (Umana et al., 2013). Furthermore, injection of anti-cholinesterase inhibitors in formalin tests in rats was found to produce anti-nociceptive effect (Yoon et al., 2003). Blockage of muscarinic receptors also has been shown to reverse anti-nociceptive activities of anti-cholinesterase inhibitors (Mojtahedin et al., 2009). Therefore, MECN may exhibit its analgesic property by acting on cholinergic receptors or inhibiting acetylcholine esterase.

There are several studies proving that flavonoids in plant extracts have anti-inflammatory and analgesic effects (Onasanwo et al., 2016, Deng et al., 2011, Saeed et al., 2010). It has also been demonstrated that plant products with alkaloids, tannins, saponins, and flavonoids have anti-inflammatory and analgesic properties (Larkins and Wynn, 2004, Fernanda et al., 2002, Rajnarayana et al., 2001). It can therefore be suggested that the anti-inflammatory and analgesic properties of MECN are produced by its phytochemical contents.

## 5 Conclusion

MECN was found to have analgesic and anti-inflammatory properties in a dose dependent manner. 200 mg/kg of MECN gave a better performance than aspirin in formalin-induced paw licking test. The analgesic effect of MECN seems to be mediated through cholinergic pathway.

## Conflict of interest

Authors have declared that no competing interest exist.
